# Different Behavioral Experiences Produce Distinctive Parallel Changes in, and Correlate With, Frontal Cortex and Hippocampal Global Post-translational Histone Levels

**DOI:** 10.3389/fnint.2018.00029

**Published:** 2018-07-19

**Authors:** Marissa Sobolewski, Garima Singh, Jay S. Schneider, Deborah A. Cory-Slechta

**Affiliations:** ^1^Department of Environmental Medicine, University of Rochester Medical Center, Rochester, NY, United States; ^2^Department of Pathology, Anatomy and Cell Biology, Thomas Jefferson University, Philadelphia, PA, United States

**Keywords:** behavior, FI schedule, forced swim, restraint stress, global histone modification, frontal cortex, hippocampus, epigenetics

## Abstract

While it is clear that behavioral experience modulates epigenetic profiles, it is less evident how the nature of that experience influences outcomes and whether epigenetic/genetic “biomarkers” could be extracted to classify different types of behavioral experience. To begin to address this question, male and female mice were subjected to either a Fixed Interval (FI) schedule of food reward, or a single episode of forced swim followed by restraint stress, or no explicit behavioral experience after which global expression levels of two activating (H3K9ac and H3K4me3) and two repressive (H3K9me2 and H3k27me3) post-translational histone modifications (PTHMs), were measured in hippocampus (HIPP) and frontal cortex (FC). The specific nature of the behavioral experience differentiated profiles of PTHMs in a sex- and brain region-dependent manner, with all 4 PTHMs changing in parallel in response to different behavioral experiences. These different behavioral experiences also modified the pattern of correlations of PTHMs both within and across FC and HIPP. Unexpectedly, highly robust correlations were found between global PTHM levels and behavioral performances, suggesting that global PTHMs may provide a higher-order pattern recognition function. Further efforts are needed to determine the generality of such findings and what characteristics of behavioral experience are critical for modulating PTHM responses.

## Introduction

The importance of behavioral history to the nature of subsequent behavioral and brain function has long been recognized (Barrett, 1986; Pattij et al., 2004; Marchner and Preuschhof, 2018) and can include differential behavioral responses to subsequent drug treatments, or even the maintenance of such seemingly incongruous behavior as response-produced shock that can emerge following specific behavioral histories (Byrd, 1972). In children, intervention programs such as Head Start, have been shown to result in long-term benefits in school achievement, grade retention, special education placement, and social adjustment (Barnett, 1995). Parallels exist in animal models using environmental enrichment. Such enrichment is achieved in several different forms, both social and physical, with multiple reports showing its ability to produce beneficial effects (Hirase and Shinohara, 2014; Kelly, 2015; Kondo, 2017). Early adverse behavioral experiences also have persistent and profound damaging effects, both cognitive and emotional. Children living in poverty, for example, have reduced gray matter volume in the frontal and temporal cortex and in hippocampus (HIPP) (Brito and Noble, 2014; Hair et al., 2015; Noble et al., 2015). Early institutionalization or being raised in an orphanage, as deprivation-related adversities, resulted in developmental delays in social, behavioral and cognitive domains (Chugani et al., 2001; Johnson et al., 2006). Some studies have even suggested that such effects may be intergenerational (Galler and Rabinowitz, 2014). The long-lasting temporal influence of behavioral experience has invoked a variety of mechanistic explanations, most notably epigenetic mechanisms.

Indeed, behavioral experiences do influence brain epigenetic profiles (Szyf et al., 2016). Glucocorticoid receptor gene methylation was found to be higher in blood of adolescents that had experienced either childhood trauma or stressful life events (van der Knaap et al., 2014). Similar findings have emerged from studies of abused or neglected children (Guillemin et al., 2014; Weder et al., 2014; Romens et al., 2015; Turecki and Meaney, 2016). Living in poverty during childhood, with its adverse conditions, produces differences in blood profile DNA methylation profiles (Borghol et al., 2012). Early child abuse leads to greater DNA methylation of the hippocampal glucocorticoid receptor (McGowan et al., 2009). In animal models, maternal deprivation in non-human primates produced widespread changes in both blood and brain DNA methylation patterns (Provençal et al., 2012), as did hierarchical status in non-human primates (Tung et al., 2012). In rodents, studies of maternal separation and differences in maternal care alter hippocampal DNA methylation (Turecki and Meaney, 2016). Although much of the current focus has been related to the epigenetic consequences of adverse experiences, studies also show that positive reinforcement based learning experiences likewise alter epigenetic marks (Day et al., 2013). Thus, epigenetic marks, through dynamic control of gene transcription, essentially frame the parameters within which brain and behavioral function can occur. It has been suggested that the trajectory of behavioral experiences that vary in both intensity and quality, may further modify brain epigenetic marks, essentially assimilating an individual’s behavioral experience and reframing the boundaries of subsequent behavioral and brain function (Szyf and Bick, 2013). Such findings likely contribute to the repeated reports of epigenetic differences in monozygotic twins (Fraga et al., 2005; Nipa et al., 2009; Wong et al., 2010; Chiarella et al., 2015).

However, it is unknown how, and to what extent, different types of behavioral experiences influence the epigenome or what characteristics of those experiences are critical to subsequent epigenetic changes that shape the parameters of future brain and behavioral function. Comparisons of epigenetic profiles in response to differing behavioral experiences are limited. One study contrasted the impact of histone deacetylase (HDAC) inhibitors on bees that had been trained to discriminate between a rewarding odor stimulus (i.e., a positive conditioned stimulus; limonene) vs. an aversive odor stimulus (i.e., a negative conditioned stimulus; natural vanilla) as conditioned stimuli predictive of subsequent access to sucrose vs. saturated NaCl solutions, respectively. It was found that HDAC inhibitors impaired discrimination memory for the aversive stimulus, but did not impact the positive reward-based conditioning, despite the fact that both were forms of learning. Additional manipulations confirmed that these observations did not reflect differences in sucrose sensitivity or locomotor activity (Lockett et al., 2014).

Such studies demonstrate that differing characteristics of the behavioral experience influence subsequent epigenetic profiles, gene expression and cellular function. Understanding the controlling characteristics is critical to advancing behavioral epigenetics and to the use of behavioral experiences as intervention strategies to reverse or reprogram epigenetic changes induced by early adversity (Szyf et al., 2016). To evaluate the role of different types of behavioral experience, the current study examined differences in epigenetic profiles in mice that had been subjected to a fixed interval (FI) schedule of food reward which consisted of earning food rewards via a lever press response that were available after FIs of time, or to a single episode of forced swim followed by a single episode of restraint stress, or to no explicit behavioral experience (remaining in the home cage).

Gene transcription is controlled by a heritable triad of epigenetic mechanisms, including gene methylation, post-translational refinement by non-coding RNAs, chromatin remodeling and histone modifications. Previous work focused on determinations of gene and site-specific changes in epigenetic methylation patterns associated with behavioral experience has clearly advanced the understanding of the molecular mechanisms by which environmentally induced epigenetic changes influence gene expression and consequent function. However, such methods ignore the network-based function of brain. A recent study of *in vivo* epigenetic imaging of HDACs found them to be concurrently highly expressed in multiple brain regions in humans, with differences between gray and white matter as well as between cortical and subcortical gray matter (Wey et al., 2016). This is not surprising given that networked connectivity is a central feature of the brain and its operation is predicted by clustered gene expression hubs, with networked, connected regions of brain exhibiting similar transcriptional profiles (Fulcher and Fornito, 2016; Xu et al., 2016; Forest et al., 2017). This suggests that epigenetic influences on gene expression connected with specific behavioral experiences may likewise be clustered, and that the brain must ultimately integrate this aggregate information in responding to behavioral experiences (Greenspan, 2001; French and Pavlidis, 2011; Wolf et al., 2011; Ji et al., 2014; Hawrylycz et al., 2015; Richiardi et al., 2015; Fulcher and Fornito, 2016; Forest et al., 2017). Given the networked nature of brain function and the epigenetic consequences of behavioral experience, this study also sought to explore whether global PTHM levels could act as an integrated signal of epigenetic modifications, as they have previously been shown to predict behavioral differences (Bousiges et al., 2010; Nesbitt et al., 2014). Global post-translational histone modification (PTHM) levels [H3K9ac, H3K4me3 (activating marks) and H3K9me2 and H3K27me3 (repressive marks)] were measured in HIPP and frontal cortex (FC) following differential behavioral experience. As evidenced by distinctive patterns of correlations between PTHMs, both within and between brain regions, such data may suggest “biomarkers” or epigenetic/genetic signatures (Kubota, 2016) for subsequent classification of behavioral events and/or directions for future behavioral studies, including shifts in gene profiles and chromatin dynamics shaped by such changes in total histone modifications.

## Materials and Methods

### Animals

Adult C57Bl6 mice (Jackson Labs, Bar Harbor, ME, United States) that had been pair-housed by sex since weaning were randomly assigned at approximately 60 days of age to receive exposure to one of three behavioral experience conditions as described below. All three behavioral experience groups have no more than 1 pup/sex from any dam, so as to mitigate any litter specific effects within behavioral experience groups. Following the completion of all behavioral experience, FC and HIPP were extracted from mice for subsequent molecular analyses. Procedures used in this study had approval of the Institutional Animal Care and Use Committees at the University of Rochester, and all mice used in this study were treated humanely and with regard for alleviation of suffering.

### Behavioral Experience

#### Fixed Interval (FI) Schedule Controlled Behavioral Experience

Fixed Interval schedule controlled behavioral experience was comprised of 30 behavioral test sessions (5 days per week, M-F) in which reinforcement (food reward; 20 mg food pellet; PJ Noyes) was programmed according to a FI 60 s schedule of reinforcement (Cory-Slechta et al., 1985, 1996, 1997). A prototypical behavioral pattern that is widespread across species emerges from this behavioral paradigm. FI test sessions were undertaken in operant chambers (Med Associates, Model ENV-307W) that were housed in cabinets that included sound-attenuation by white noise generators and fans for ventilation. The back wall of the chamber had three response levers configured horizontally, with a dual liquid dipper and food pellet dispenser delivery on the front (opposite) wall. The FI schedule requires a single lever press on the designated active lever (left lever) after the specified time interval (60 s) ends, which produces food delivery and starts the subsequent 60 s interval. Responses on any lever during the 60 s interval do not have any programmed consequences. FI test sessions were 30 min in duration. Standard performance measures of FI behavior were used were overall rate (defined as the total number of responses/total session time), the postreinforcement pause (PRP) time (defined as the time from the beginning of the 60 s interval to the occurrence of the first response), run rate (defined as the total number of responses/total time minus the PRP time) and interresponse time (IRT) or mean time between responses as previously described (Cory-Slechta et al., 1998).

#### Forced Swim-Restraint Stress (FS-RS) Behavioral Experience

Forced Swim-Restraint Stress (FS-RS) behavioral experience was comprised of an initial exposure to one 5 min session of forced swimming followed 1 week later by a single 30 min episode of restraint (immobilization) stress. For the forced swim exposure, mice were placed into a bucket of water that precluded escape, and that did not allow mice to use their tail to balance. The following week, a single 30 min immobilization restraint stress was carried out in which mice were placed in a restrictive plastic tube measuring 4.5″ in length and 1.1″ in width. Measures of forced swim included mean float duration, total float duration, mean duration swimming, and total duration swimming. There was no particular rationale for the order of these events, as both were considered highly salient.

#### No Behavioral Experience

No behavioral experience consisted of mice remaining in the home cages for the entire duration of behavioral experience afforded to the other groups.

Since the FI schedule of food reward requires food-motivation, all mice in all behavioral experience groups underwent caloric restriction beginning at approximately 50 days of age that maintained body weights at 90% of ad-lib weights; this continued for the duration of the experiment. Body weights were collected daily from M to F. Brains from all three experience groups were collected 1 week following completion of the FI schedule behavioral testing. For this purpose, consistent sections of FC and HIPP were dissected from fresh brain tissue. As FS-RS behavioral experience was completed before FI behavioral experience, a longer overall post-behavior experience time (approximately 1 week) elapsed for this group, resulting in some differences in overall timing between these experiences and global PTHM assessment. However, extending the duration of the FS-RS experience (i.e., repeated exposures) would also have led to habituation, and thus potentially diminished the salience of the differences in behavioral experience conditions (Babb et al., 2014; McInnis et al., 2015; Sadler and Bailey, 2016).

### Measurement of Global Post-translational Histone Modifications Levels

A random subset of 5 of the 12 animals from each sex/behavioral experience group were used for these analyses. FC and HIPP were dissected from each of those 5 brain brains and homogenized for subsequent analyses. Thus, sample size for PTHM expression levels was *n* = 5 for each region/sex/behavioral treatment group and all PTHM expression level analyses utilized the homogenized tissue.

#### Histone Purification

Histone Purification An Active Motif Histone Purification mini kit (Cat. No. 40026) was used to purify histones via the protocol provided by the manufacturer, with some modifications. For this purpose, the dissected FC and HIPP collected at the termination of behavioral testing (five biological replicates per sex per behavioral experience group), all obtained from the left hemisphere were homogenized in 0.3 ml extraction buffer and incubated overnight at 4°C on a rotating platform. All steps were performed at 4°C. The crude histone extract was neutralized by adding 5× neutralization buffer prior to purifying the histone proteins. Purification was done using spin columns that were provided with the kit, which were then washed three times with 0.5 ml histone wash buffer. Elution was done in 0.05 ml elution buffer. The histone proteins were then precipitated overnight using perchloric acid to a final 4% concentration, after which they were pelleted by spinning at 14,000 rpm for 1 h at 4°C. Salts were eliminated by first washing twice with 0.5 ml 4% perchloric acid, then by acetone containing 0.2% HCl and finally with acetone. Pellets were then allowed to air dry, after which they were dissolved in 0.03 ml sterile water and snap frozen histone protein extract was stored at -80°C. Histone protein concentrations were measured according to manufacturer instructions using a Qubit Protein Assay Kit (Invitrogen, Cat No. Q33212).

#### Detection of Global Post-translational Histone Modifications Levels

Detection of global PTHMs levels The Active Motif Histone H3 PTM Multiplex Kit (Cat No. 33115) was used to measure global changes in PTHM levels. the Histone H3 Total Ab-conjugated beads (Cat No. 33116) were multiplexed with the Histone PTM Ab-conjugated beads (H3K9ac Cat No. 33117, H3K4me3 Cat No. 33121, H3K9me2 Cat No. 33119, and H3K27me3 Cat No. 33125) for normalization of Histone H3 levels between different samples. A total of 150 ng of purified histones were used for each assay. Data were collected using a FLEXMAP3D instrument and analyzed using xPONENT software (Luminex Corporation). Data were normalized to show the relative amount of each PTHM in different samples.

### Statistical Analyses

Based on our prior repeated observations of sex-dependent differences in epigenetic marks (Cory-Slechta et al., 2004, 2010; Weston et al., 2014; Schneider et al., 2016), all statistical analyses were carried out separately by sex. For assessment of PTHM levels, one way analyses of variance (ANOVAs) were carried out with behavioral experience as a between groups factor (FI, FS-RS, no experience), followed by *post hoc* student’s *t*-test method tests as appropriate. Multivariate analyses were carried out to examine correlations of the global levels of PTHM marks within and across FC and HIPP. To determine whether the correlational structure changed significantly in response to behavioral experience conditions, chi square analyses were carried out across the three behavioral experience conditions, comparing the frequency of statistically significant vs. non-significant *p*-values. In addition, multivariate correlation analyses were carried out between measures of FI and of FS-RS behavioral performance with levels of global PTHMs. These were analyzed separately by sex and behavioral experience condition. For all analyses, a *p* ≤ 0.05 was considered statistically significant.

## Results

### Global PTHMs Differ in Response to Behavioral Experience: Sex and Brain-Region Dependence

**FIGURE 1:**
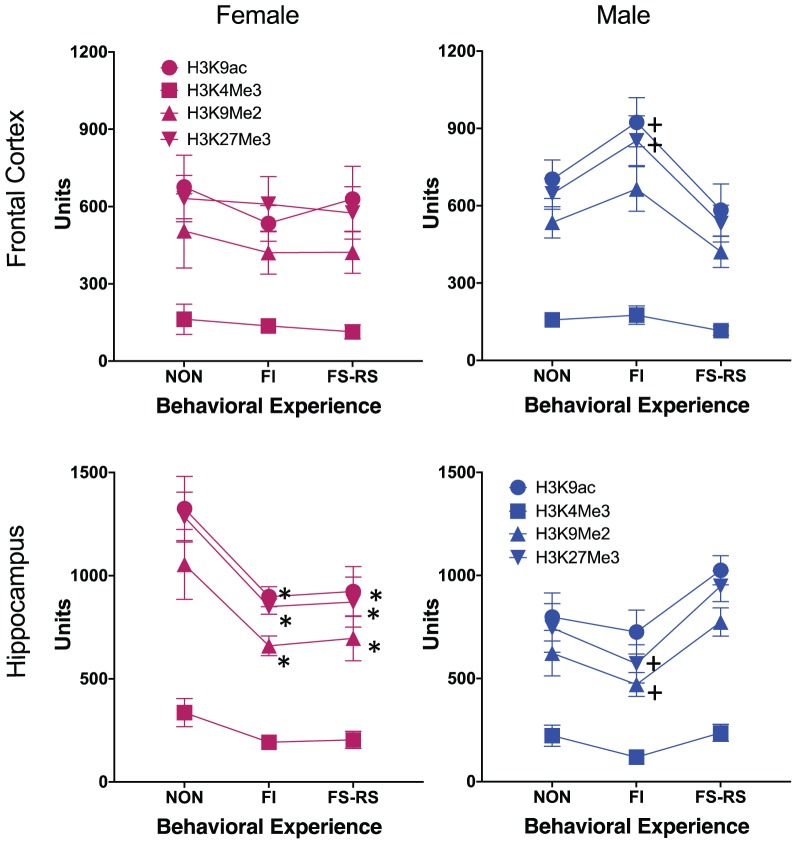
Group mean ± SE values for relative enrichment of the indicated global post-translational histone modification in frontal cortex and hippocampus (HIPP) of males and females in relation to the type of behavioral experience. Non, no behavioral experience group; POS, positive behavioral experience group; NEG, negative behavioral experience group (*n* = 12/group). ^∗^ significantly different from no behavior group; + significantly different from positive behavior group. + indicates significant difference from negative behavior group.

Global PTHM levels changed in response to behavioral experience conditions and did so differentially by brain region for each sex as shown in **Figure 1**. Male-specific differences in global PTHM levels in FC in relation to behavioral experience conditions were found for H3K27me3 [*F*_(2,14)_ = 4.43, *p* = 0.036] which reflected the higher levels of this mark in mice that had FI behavioral experience (*p* = 0.0124), compared to those with FS-RS behavioral experience, with a similar trend (*p* = 0.061) evident for H3K9ac in males. No significant differences were found for females. In HIPP, differences were particularly pronounced in females, with marked reductions (30–49%) in levels of all 4 PTHMs in groups that had either FI or FS-RS experiences [H3K9ac = *F*_(2,14)_ = 7.75, *p* = 0.008; *post hoc t*-test *p*-values of 0.0061 and 0.0045, respectively, H3K4me3 = *F*_(2,14)_ = 4.38, *p* = 0.04], with reductions of 46–49% (*p* = 0.02 and 0.028, respectively, from non-behavioral control); H3K9me2 = [*F*_(2,14)_ = 8.55, *p* = 0.006; *p* = 0.003 and 0.005, respectively, H3K27me3 = *F*_(2,14)_ = 9.12, *p* = 0.005]. In males, the trend in HIPP was for reduced levels (approximately 10–48%) in the FI behavioral experience groups relative to both non-behavioral and FS-RS behavioral experience groups for 3 of the 4 PTHMs examined, i.e., the opposite pattern of what was observed in FC. These trends in HIPP approached significance for H3K27me3 [*F*_(2,14)_ = 3.81, *p* = 0.052] and for H3K9me2 [*F*_(2,14)_ = 3.41, *p* = 0.067].

### Correlations of Global PTHM Levels Within and Across Brain Regions in Relation to Behavioral Experience Condition

**FIGURE 2:**
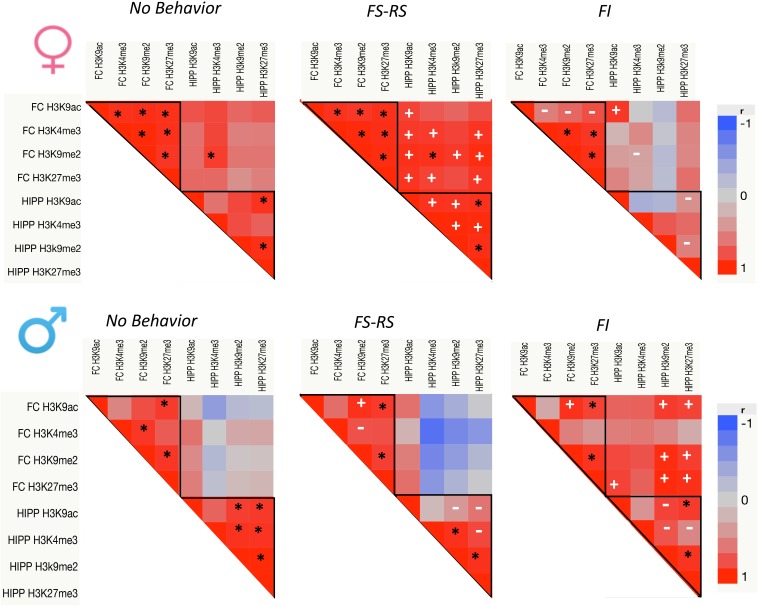
*p*-Value heat maps from multivariate correlation analyses across all PTHM levels in both FC and HIPP for each behavioral experience group of females and males (*n* = 5 per group). Black asterisks show significant correlations preserved across all three behavioral conditions; white plus marks show new correlations within the positive or negative behavioral experience groups; white dashes indicate significant correlations that were lost following either positive or negative behavioral experience relative to no behavior control.

**Figure 1** suggested that global levels of these 4 PTHM generally changed in parallel in response to a specific behavioral experience. Consequently, correlations of global PTHM levels within and across brain regions were examined in relation to behavioral experience conditions separately for each sex (**Figure 2**). Both within and across region correlations differed by behavioral experience for each sex.

For females (top row), under conditions of no behavioral experience, correlations of PTHMs largely occurred within a brain region, i.e., within FC and within HIPP regions, with only a single correlation across regions. Specifically, correlations between PTHMs were more pronounced within FC (*r* range = 0.92–0.995) with a more modest correlation profile within HIPP, and with a single inter FC-HIPP correlation. Following FS-RS experience, intra FC global PTHM correlations remained as in the no behavior conditions, while this experience significantly enhanced the number of within-HIPP PTHM level correlations (intra-FC correlations *r* range = 0.92–0.98 and intra-HIPP *r* range = 0.93–0.987). FS-RS experience also markedly increased the numbers of inter FC-HIPP correlations (*r* range = 0.865–0.974 for PTHMs). After FI behavioral experience, the number of within FC global PTHM correlations was reduced relative to no behavior control, particularly for H3K9ac, and within HIPP global PTHM correlations were eliminated. While the one inter FC-HIPP correlation seen under no behavioral experience conditions was also eliminated after FI experience, a new correlations between FC and HIPP global H3K9ac emerged. Chi-square analyses confirmed that the frequency of significant correlations of PTHM levels differed significantly in relation to behavioral experience (*x*^2^ = 48.98, *p* < 0.0001) with all three groups differing significantly from each other in frequency.

In males (bottom row) with no behavioral experience, global PTHM correlations were found within both HIPP and FC (*r* range = 0.887–0.996), but no inter FC-HIPP correlations occurred. After FS-RS behavioral experience, numbers of global PTHM level correlations were generally retained within FC, albeit in a different profile, while global PTHM correlations within HIPP were diminished relative to the no behavior condition, and no significant inter FC-HIPP correlations were found. After FI behavioral experience, within FC global PTHM correlation profiles were analogous to those seen after FS-RS experience, while within HIPP correlations were reduced. FI experience produced a marked increase in inter FC-HIPP correlations particularly between HIPP H3K9me2 and H3K27me3 with global levels of FC H3K9ac, H3K9me2 and H3K27me3. Chi-square analyses of global PTHM changes indicated a trend toward significant differences in the frequency of significant correlations by behavioral condition (*x*^2^ = 4.56, *p* < 0.102) which reflected the difference between the FI vs. RS-FS behavior groups.

### Correlations of Global PTHM Levels With Measures of Behavioral Experience

**FIGURE 3:**
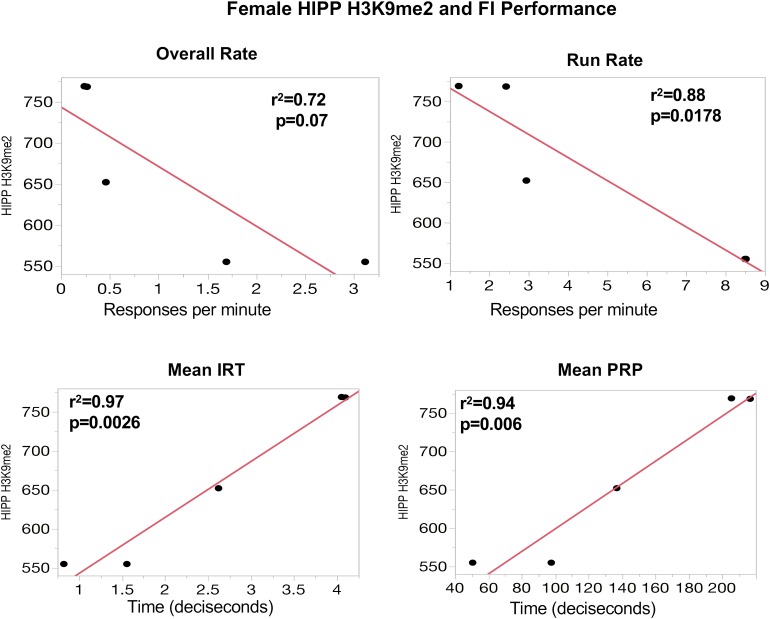
**(Top)** Correlations between measures of FI performance in session block 6–10 and global HIPP levels of H3K9me2 in females. The *y*-axis depicts levels of HIPP H3K9me2 expression and the *x*-axis depicts response rate values (responses/minute) for overall rate and run rate, or time (in deciseconds) for mean interresponse time (IRT) or postreinforcement pause (PRP) as indicated. The red line through each plot depicts the linear fit, and *r*^2^- and *p*-values are indicated for each plot.

**FIGURE 4:**
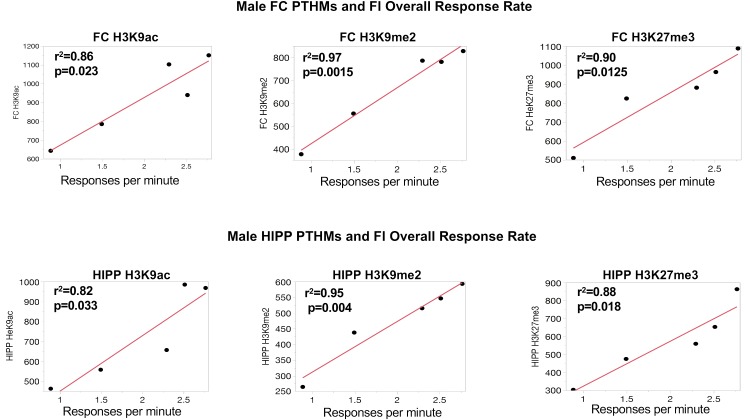
Correlations between measures of FI overall response rates and global levels of H3K9ac, H3K9me2, and H3K27me3 in FC and HIPP, respectively. The red line through each plot depicts the linear fit, and *r*^2^- and *p*-values are indicated for each plot.

To further support the behavioral relevance of global PTHM measures, examination of correlations of global PTHM levels with measures of behavioral performance were carried out. Multiple significant correlations were found with very high *r*^2^-values. For example, in females (**Figure 3**), strong correlations between HIPP PTHM H3K9me2 occurred with multiple measures of performance on the FI schedule, particularly during the early sessions (period of learning of the FI schedule parameters). Specifically, higher overall rates and run rates on the FI schedule were associated with lower levels of HIPP H3K9me2, and correspondingly with shorter IRTs and PRP times. *r*^2^-values for these correlations were notable, and ranged from 0.72 to 0.97. Measures of FI performance in males (**Figure 4**) correlated with global PTHM levels in both FC (middle row) and HIPP (bottom row). Specifically, increasing levels of H3K9ac, H3K9me2, and H3K27me3 in both FC and HIPP were correlated with higher overall response rates on the FI schedule, with *r*^2^-values that ranged from 0.82 to 0.95.

**FIGURE 5:**
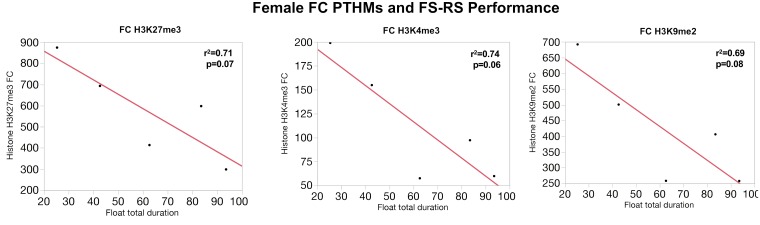
Correlations between measures of total duration floating in the FS paradigm and global levels of FC H3K27me3, H3K4me3, and H3K9me2 in females. The red line through each plot depicts the linear fit, and *r*^2^- and *p*-values are indicated for each plot.

**FIGURE 6:**
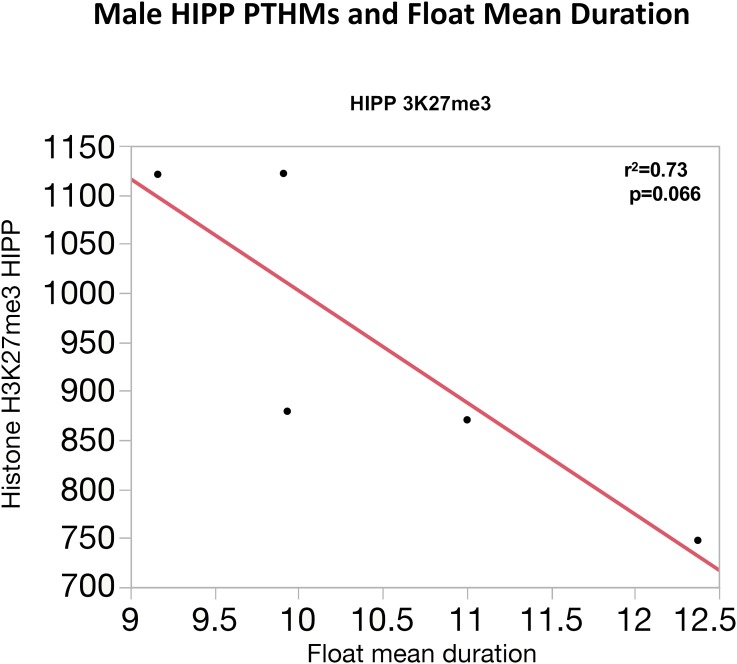
Correlation between mean floating duration time in the FS paradigm and global levels of HIPP H3K27me3 in males. The red line through each plot depicts the linear fit, and *r*^2^- and *p*-values are indicated for each plot.

Similarly, correlations between global PTHM levels and measures of FS-RS were observed, although of marginal significance. **Figure 5** depicts correlations between FC PTHMs and FS-RS performance, with increases in total float duration times marginally correlated with reductions in levels of FC PTHMs H3K27me3, H3K4me3, and H3K9me2, with *r*^2^-values ranging from 0.69 to 0.74. In the case of males (**Figure 6**), a marginally significant correlation was observed in which increases in mean float duration were associated with reductions in HIPP PTHM H3K27me3 with an *r*^2^-value of 0.73; similar trends were evident for other PTHMs in both males and females.

## Discussion

This study examined the influence of contrasting behavioral experiences, specifically food-rewarded learning performance on an FI schedule and single episodes of two different and salient stressors, forced swim followed by restraint stress, or to no explicit behavioral experience, on FC and hippocampal global levels of two global activating (H3K9ac and H3K4me3) and two global repressive (H3K9me2 and H3K27me3) PTHMs. Three overall findings from this study deserve mention, two of which were quite unexpected. First, the specific type of behavioral experience was shown to be a critical determinant of the subsequent global PTHM profiles, and these evidenced both brain region and sex differences. Second, these global PTHMs appeared to be changing in parallel in relation to the specific behavioral experience, i.e., with similar effects on levels of all 4 PTHMs in each behavioral condition. Third, even though these were global levels of histone modifications (and thus not indicative of changes at specific genes or sites on genes), they nevertheless showed robust correlations with measures of behavioral performance.

The results clearly demonstrate the importance of behavioral context and experience to subsequent PTHM modifications. For example, males showed trends toward increases in PTHM levels in FC, but decreases in HIPP after FI experience, whereas changes evident after FS-RS behavioral experience were not significant (**Figure 1**). In females, differences in global PTHM levels were restricted to HIPP, and comprised of equivalent reductions of PTHM levels in groups with either FI or FS-RS experience. Reports of direct comparisons in PTHM levels following differing behavioral experiences, and/or comparisons to a no-behavioral control group are limited to date. In one such study that did involve such comparisons in bees, systemic administration of HDAC inhibitors impaired olfactory discrimination memory for an aversive stimulus, but not for a rewarding odor, effects shown not to reflect differences in sucrose sensitivity or locomotor activity (Lockett et al., 2014). Although based on different outcome measures, both were measures of learning and show that the nature of the behavioral experience was important to the consequent epigenetic outcome. Another reported study (Bousiges et al., 2010) involved rats that had either undergone water maze learning that included a constant location visible platform for escape was possible or a water maze in which the platform was relocated for each trial. Findings showed increases in dorsal hippocampal global H2B and H4 acetylation levels in the hidden platform condition, but not the relocated platform condition., while an additional experiment revealed markedly increased H3 acetylation after the variable platform experience in comparison to levels obtained from rats that remained in their home cage.

The current findings also show clear brain region and sex-dependent differences in epigenetic profiles following behavioral experience, confirming many previous reports (McCarthy and Nugent, 2015). For example, exposure to subchronic variable stress in rodents resulted in significant sex-specific differences in *Dnmt3a* gene expression levels in nucleus accumbens (Hodes et al., 2015). Genome-wide assessment of H3K4me3 in adult mice revealed differences in its expression by sex in 248 genes and loci (Shen et al., 2015). Prenatal stress differentially altered gene expression and epigenetic regulation in HIPP and FC of rats in a sex-dependent manner (Van den Hove et al., 2013). Further, levels of histone modifications were found to differ by sex in neonatal mouse brain (Tsai et al., 2009). Thus, the sex differences observed here both confirm and extend those findings.

With respect to brain region differences, studies to date have often focused on single brain regions. Yet, regionally specific responses in histone marks to behavioral experience are increasingly being identified. For example, in an object recognition task, PTHM level profiles and activity over time differed significantly in HIPP and prefrontal cortex in mice, as did expression of the learning-related gene zif268 (Graff et al., 2012). Furthermore, in a report consistent with our findings, levels of histones H2B, H3, and H4 acetylation were found to differ both within region and across regions (HIPP and dorsal striatum) in mice trained in spatial vs. cued water maze learning (Dagnas and Mons, 2013).

Remarkably, global PTHM levels appeared to be changing in parallel in relation to behavioral experience, i.e., with generally similar effects on levels of all 4 PTHMs within a brain region in each behavioral condition. Furthermore, the significance of those changes was clearly demonstrated by their very robust correlations with measures of behavioral performance. Such findings demonstrate that both total levels of histone modifications, in addition to studies that suggest that effects are gene/loci specific (Tsankova et al., 2004; Park et al., 2017), are needed to understand the complex suite of epigenetic shifts occurring following behavioral experience. It seems intuitive that the understanding behavioral function, which involves the integrated and immediate activity of networks of regions within the brain, requires an iterative research approach of integrative/higher-order and gene-specific measures. Studies show brain network connectivity to be a central feature of its operation that can actually be predicted by clustered gene expression profiles across regions (Greenspan, 2001; French and Pavlidis, 2011; Wolf et al., 2011; Ji et al., 2014; Whitney et al., 2014; Hawrylycz et al., 2015; Richiardi et al., 2015; Fulcher and Fornito, 2016; Xu et al., 2016; Forest et al., 2017). These gene clusters may be best represented by a global or total measurement of histone modifications across the genome (Nesbitt et al., 2014). Epigenetic changes, even while differing at local sites, nevertheless occur simultaneously in multiple brain regions, as shown by *in vivo* imaging of HDACs in humans (Wey et al., 2016). In fact, our findings of robust correlations of behavioral performance with measures of global PTHMs are similar to a previous report of correlations of locomotor activity with global levels of hippocampal acetylation which differentiated animals exhibiting low vs. high levels of activity. Here too correlations were observed with global PTHMs, albeit not generally as robust as those shown here, likely reflecting the fact that brains were obtained 4 months after behavioral assessment (Nesbitt et al., 2014). One implication of our findings would be that such global histone marks could serve as “biomarkers” or indicators (Kubota, 2016) of past behavioral experience which could provide a far more cost-effective approach to delineating the full spectrum of individual PTHMs by gene, region and cell type (Henikoff and Shilatifard, 2011).

One question raised by these findings is what role such global changes could play in brain. Nesbitt et al. (2014) suggested that such global changes may represent a potential mechanism to regulate cellular plasticity in brain, and further, that reports identifying gene-specific changes in chromatin marks could be due to primary or upstream changes in total levels of chromatin marks that then facilitate the progression or trajectory of gene expression programs. Extending this idea, a higher order level of control is suggested, i.e., a type of “pattern recognition” by the brain controlling gene expression, as ultimately the brain must integrate more localized epigenetic changes across systems/networks. Evidence for importance of global histone marks in predicting multiple types of cancer, as well as its progression and response to chemotherapy is already well established (Ebrahimi et al., 2013; Karczmarski et al., 2014; Ellinger et al., 2016; Zhao et al., 2016; Ngollo et al., 2017).

The current findings also raise questions as to what specific characteristics/processes of behavioral experiences differentiate their epigenetic consequences, and whether similar behavioral domains (e.g., learning of any kind) produce corresponding profiles of epigenetic changes. To date, studies of behavioral epigenetics have often tended to focus on single behaviors (e.g., learning, drug abuse, or stress) and frequently examine that behavior using a very limited range of paradigms. For example, studies of epigenetic coding of learning/memory have primarily relied on the use of fear conditioning (Day and Sweatt, 2011; Sweatt, 2016). This raises the question of whether reported epigenetic changes in such studies are purely “learning”-related, i.e., generalizable across different learning paradigms, or reflect specific components of the paradigm used, e.g., shock-based learning. Would positive reinforcement-based learning paradigms produce the same epigenetic profiles as negative reinforcement based learning paradigms? One limitation of our study was the differences in timing between the end of behavioral testing and consequent determination of PTHMs, which was longer in the FS-RS as compared to the FI behavioral experience group by 1 week, providing an opportunity for more PTHM changes in that condition post-testing. This could account for the lesser evidence for correlations between global PTHM and FS-RS experience as compared to FI-based correlations. Even with this limitation, however, overall behavioral experience conditions of the groups were markedly different and still evidenced in differences in global PTHM levels and in correlations of these global PTHMs with behavior. Further, correlations between global acetylation levels and locomotor activity survived a 4 months post-behavioral assessment time frame in the study by Nesbitt et al. (2014).

These global differences in PTHM profiles would seem to be consistent with the potential for differential types of networked activity following FI vs. FS-RS vs. no behavioral experience. While it might be asserted that some within-animal correlations would certainly be expected, this seems an unlikely explanation for the patterns observed here for at least two reasons. First, it would not explain why all PTHMs were not uniformly or close to uniformly correlated. Nor would it explain the fact that marked differences in the PTHM correlation profiles occurred in response to different types of behavioral experience. Future studies of gene specific genome wide and transcriptomic effects, using ChiP-sequencing and RNA sequencing methods, of these behavioral experiences will be required to fully elucidate the influence of these behaviors on the neural connectome at a gene-specific level. Further, while recognizing that correlational analyses does not recapitulate direct molecular mechanistic function or indicate causation, advancing the understanding of behavioral epigenetics will also require systematic assessment of network-based outcomes to answer the questions related to how brain ultimately integrates the localized molecular changes to particular patterns of behavioral experiences.

## Author Contributions

DC-S, JS, and MS conceived the experiments. MS and DC-S oversaw the behavioral assessments and all statistical analyses and GS and JS contributed in histone determinations. DC-S wrote the manuscript with input from JS, MS, and GS.

## Conflict of Interest Statement

The authors declare that the research was conducted in the absence of any commercial or financial relationships that could be construed as a potential conflict of interest.
